# Direct Intrahepatic Portosystemic Shunt in Budd-Chiari Syndrome: A Case Report and Review of the Literature

**DOI:** 10.1155/2018/9261268

**Published:** 2018-08-23

**Authors:** V. Chandra, E. Wajswol, M. Shahid, A. Kumar, S. Contractor

**Affiliations:** Department of Radiology, Rutgers New Jersey Medical School, Newark, NJ, USA

## Abstract

Transjugular intrahepatic portosystemic shunt (TIPS) is an alternative interventional procedure used to manage refractory Budd-Chiari syndrome (BCS) when conservative medical therapy has failed. However, TIPS is not always technically successful because of hepatic vein thrombosis and inability to catheterize the hepatic veins. In these situations, direct intrahepatic portosystemic shunt (DIPS) with access to the portal vein from the IVC has been shown to be a viable alternative that may ameliorate portal hypertension in these patients. Typically, DIPS involves the use of transabdominal ultrasound to target the portal vein. Herein a case in which a 39-year-old female underwent DIPS without the use of ultrasound guidance is presented. Instead, a hepatic venogram generated using collateral circulation was used to opacify and guide access to the portal vein.

## 1. Introduction

Budd-Chiari syndrome (BCS) is associated with occlusion of hepatic venous outflow by thrombosis or structural compression at the level of the main hepatic vein or the extrahepatic segment of the inferior vena cava [1]. Initial management of BCS is usually medical (nonoperative/noninterventional), with the most common intervention for BCS nonresponsive to medical management being transjugular intrahepatic portosystemic shunt (TIPS). However, this is not always technically successful because of hepatic vein thrombosis and inability to catheterize the hepatic veins. In these situations, direct intrahepatic portosystemic shunt (DIPS) with access to the portal vein from the IVC is a viable alternative that may ameliorate portal hypertension in these patients [2–5]. This procedure generally involves the use of transabdominal ultrasound or endovascular ultrasound to accurately identify and access the portal vein. If the portal vein can be identified by catheterizing the collateral vessels seen in BCS, the portal vein may be targeted from the IVC without the use of ultrasound. Herein is presented a case in which a 39-year-old female underwent DIPS after the portal vein was localized utilizing the opacified collateral circulation as a roadmap.

## 2. Clinical History

A 39-year-old Hispanic female with no significant past medical history presented to an outside hospital with complaints of nausea, vomiting, and abdominal pain. The patient's presentation and workup was consistent with decompensated liver cirrhosis. The patient was started on diuretics and underwent multiple paracentesis for ascites, as well as being treated for spontaneous bacterial peritonitis (SBP). Primary prophylaxis with propranolol was initiated after esophagogastroduodenoscopy (EGD) showed grade II esophageal varices and portal gastropathy. The patient was also found to have a large deep vein thrombosis (DVT) extending from the IVC to the right common and external iliac veins and was started on heparin drip for anticoagulation. Cirrhosis workup for chronic liver disease was negative, including viral and autoimmune etiologies. Polycythemia vera or other chronic myeloproliferative disorder was suspected given patients high peripheral red blood cell count (RBC 6.15/uL, Hemoglobin 16.0 g/dL, and Hematocrit 48.9%). Further workup, including Factor V Leiden, Factor II prothrombin gene mutation, antiphospholipid antibody syndrome, and JAK2 mutation, was negative and biopsy of the liver showed complete cirrhosis without marked steatosis. Despite receiving maximal medical therapy, the patient continued to have significant ascites requiring multiple large-volume paracentesis. Given the refractory ascites and the presence of a DVT, Budd-Chiari syndrome was suspected and TIPS placement was attempted at the outside hospital. TIPS placement was abandoned after the hepatic veins could not be catheterized due to narrowing vs. occlusion.

The patient was transferred to this author's hepatology service for liver transplant workup and potential surgical shunt placement. On initial presentation the patient denied abdominal pain, nausea, vomiting, diarrhea, confusion, and weakness. The patient appeared jaundiced but in no acute distress, with scleral icterus noted on HEENT exam. On neurological exam, the patient was alert and oriented to person, place, and location, with no asterixis noted. Examination of the abdomen yielded distension, a positive fluid wave, and right upper quadrant tenderness to palpation. On presentation, the patient had a Child-Pugh Score of 7, model end-stage liver disease (MELD) score of 23, and a class I Rotterdam score of 1.04.

## 3. Imaging Review

Doppler ultrasound of the abdomen demonstrated hepatomegaly, hepatic parenchyma consistent with cirrhosis, a patent portal vein and IVC, significant ascites, and splenomegaly. Hepatopetal flow was noted in the main, right, and left portal veins. The hepatic vein could not be visualized. CT imaging displayed classic features of BCS, including heterogenous “starry sky” liver parenchyma, lack of visualization of the hepatic venous trunks, and an enlarged caudate lobe. CT imaging was also significant for changes consistent with portal hypertension. In view of this picture, TIPS was considered as a means of alleviating her portal hypertension.

## 4. Treatment Options/Results

**Figure 1:**
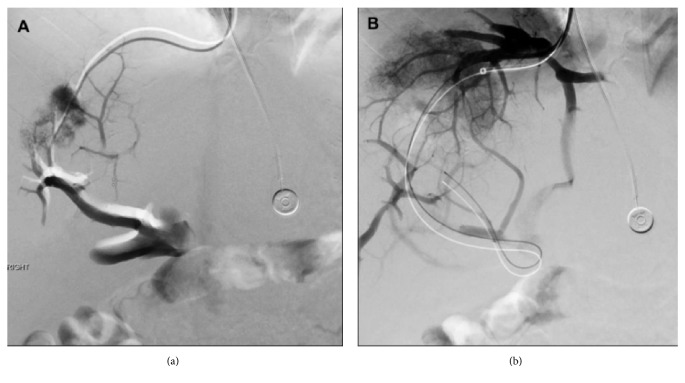
(a) Initial venography of right hepatic venous system. Note parenchymal blush and opacification of the hepatic veins and portal venous system. (b) Venography following distal placement of the guidewire. Note the guidewire is now within the portal venous system. Parenchymal blush can be seen as well as opacification of the hepatic venous system and multiple collaterals leading, from the hepatic venous system to the portal system.

Under ultrasound guidance, vascular access was obtained via the right internal jugular (RIJ) vein using a 5 Fr micropuncture kit. An Amplatz wire was passed into the IVC and a 12 Fr vascular sheath was then advanced over the wire into the IVC. A venogram was performed which demonstrated the site of inflow from the hepatic veins. Utilizing a hydrophilic Glidewire and 5 Fr Berenstein catheter, attempts were made to catheterize the right hepatic vein. Venogram at this point demonstrated outflow venous obstruction with reflux of contrast into the right portal vein (RPV), shown in Figure 1(a). Angiography following further distal advancement of the guidewire revealed the presence of the guidewire within the portal system, introduced without assistance from a needle (Figure 1(b)).

**Figure 2:**
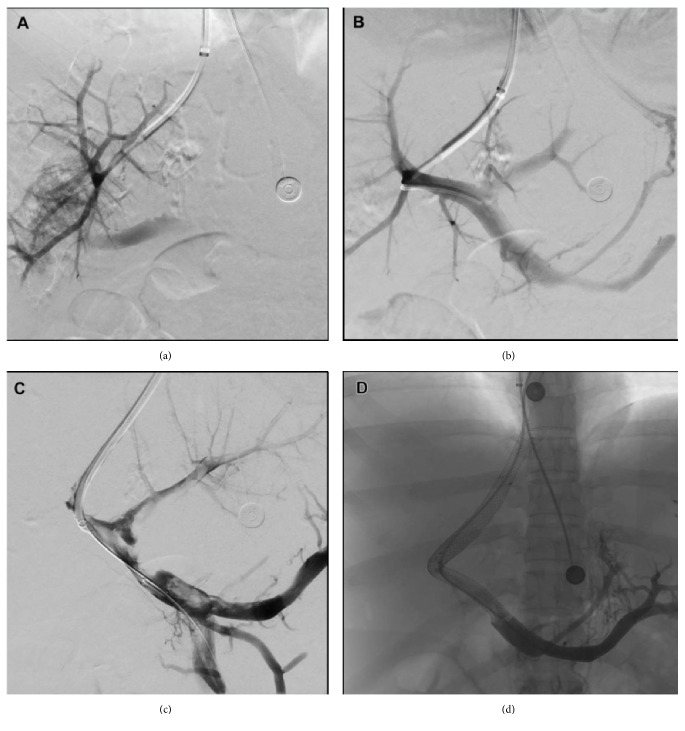
(a) Portal venography after initial needle access through DIPS. (b) Portal venography after introduction of sheath into the portal venous system. Note the presence of gastric varices. (c) Portal venography prior to creation and stenting of the DIPS. Note portal venous thrombosis disseminated throughout. (d) Unsubtracted post-DIPS and stenting portal venography. Note the absence of thrombosis and patency of the DIPS with brisk flow of contrast.

Attempts were made to catheterize the occluded hepatic veins; however, the sheath of the TIPS set could not be placed in the hepatic vein. The Glidewire was left within the portal system and the sheath was pulled back to level of the IVC. Using the Glidewire as a guide, serial balloon catheter dilations were performed without technical success. At this point, a decision to perform a DIPS was made. An exaggerated curve of the outer cannula of the TIPS sheath was made. The liver was probed with the puncture needle from the IVC just below the previously identified origin of the hepatic vein. The cannula was then rotated counterclockwise and advanced through the liver parenchyma until a portal vein was successfully accessed (Figure 2(a)).

A hydrophilic wire and catheter were then passed into the portal vein and a portal venogram demonstrated hepatic flow with evidence of prominent esophageal and gastric varices (Figure 2(b)). An Amplatz wire was then passed into the RPV; the hepatic parenchymal tract was dilated using a 6 mm angioplasty balloon. A 10 Fr introducer sheath was then passed into the RPV and a 10 mm × 8 cm Viatorr covered stent (W.L. Gore & Associates, Inc, Flagstaff, Arizona) and a second 10 mm × 98 mm Wallstent (Boston Scientific, Marlborough, Massachusetts) were placed spanning from the portal vein to the hepatic vein IVC junction. The stent was then dilated utilizing a 10 mm angioplasty balloon. Venograms prior to creation of DIPS and stenting demonstrated new partial thrombosis of the portal vein (Figure 2(c)). At this time, a 5 Fr Fogarty balloon catheter was used to sweep the thrombus and free flow in the portal vein was restored. Poststenting venogram demonstrated the absence of significant residual thrombus and excellent flow through the stented segment, with decompression of the previously visualized esophageal and gastric varices (Figure 2(d)). The patient tolerated the procedure well and with the exception of the aforementioned resolved intraprocedural thrombosis experienced no complications.

Ultrasound evaluation 2 weeks later revealed the main portal vein to have hepatopetal flow, with a flow velocity of 47.3 cm/s. The left portal vein was patent with hepatopetal flow, and the right portal vein was patent with bidirectional flow. Velocity was 92 cm/s within the proximal DIPS, 131 cm/s within the mid DIPS, and 127 cm/s within the distal DIPS.

## 5. Discussion

Budd-Chiari syndrome (BCS) is a group of disorders characterized by hepatic venous outflow occlusion, by either thrombosis or structural compression, leading to hepatovenous congestion, ischemic necrosis, and eventually cirrhosis [1]. While the progression of BCS can be fulminant, acute, or chronic, it is usually asymptomatic for a long period of time until progressing rapidly to liver cirrhosis and the development of portal hypertension [6]. If timely diagnosis and disease management are not initiated, there is a significant morbidity associated with BCS, with 1-year and 3-year untreated mortality exceeding 70% and 89%, respectively [7, 8]. The principal goal of treatment and disease management in BCS is to decrease hepatic congestion and thereby reduce the associated morbidity and mortality.

Since BCS is a pathophysiologic process that encompasses multiple etiologies, management in patients with BCS depends on clinical symptoms and anatomic considerations. The American Association for the Study of Liver Diseases recommends a stepwise approach in management of BCS, beginning with medical treatment prior to interventional approaches [9]. Since an underlying condition can be diagnosed in up to 80% of patients with BCS, appropriate medical management should be initiated promptly [10]. This most often involves anticoagulation to address the primary hypercoagulability disorder and prevent propagation of clot formation [11]. However, one study showed the use of anticoagulation alone to be successful in only 18% of patients and radiographically guided treatments were required to restore patency of the thrombosed hepatic veins [7].

Minimally invasive interventional treatment options include recanalization of hepatic veins with balloon angioplasty, hepatic vein stenting, and transjugular intrahepatic portosystemic shunt (TIPS). In China, balloon angioplasty, with or without stenting, is the most common interventional treatment employed in treating BCS and TIPS is rarely used [12, 13]. This success of balloon angioplasty in China is likely attributable to the etiology of BCS in the Asian population; BCS in the Asian population is usually associated with suprahepatic stenosis of the IVC and therefore TIPS is not therapeutic [14, 15]. This is in contrast to the pathophysiology of BCS in Western countries, as it includes typically hypercoagulable states that lead to hepatic vein occlusion [16]. Recanalization procedures in this population only provided a clinical benefit in 15% of patients [7]. Studies have also shown that placement of metal stents along with angioplasty is associated with decreased incidence of reocclusion and may in fact improve long-term survival [17–20].

Interventionalists are often conservative when considering the use of stent, as it can complicate liver transplantation should the patient require one. While angioplasty and stenting are attractive options, they seem to have a low applicability in the treatment of BCS patients in the western world, as they only prevent progression of treatment to TIPS and liver transplant in a third of patients [6, 21, 22]. Furthermore, it has been shown that among pediatric BCS patients success rates for angioplasty, hepatic vein stenting, and TIPS were 43%, 66%, and 72%, respectively [23]. A comprehensive study has not yet reported results in the adult population, and currently there are no randomized controlled trials comparing these interventional procedures.

TIPS has been shown to have a high technical success rate of 93%, with a 1- and 5-year transplant-free survival rate of 93% and 74%, respectively [24, 25]. While only 50% of patients who underwent recanalization procedures showed a clinical response, TIPS showed an increased response rate of 84% [7]. Moreover, TIPS is associated with less morbidity and mortality when compared to open surgical procedures [1, 26]. Despite the lack of RCTs, there exists some retrospective evidence suggesting that TIPS may improve survival in BCS patients who fail to respond to medical therapy [25, 27].

Despite these high success rates of TIPS among BCS patients, TIPS is sometimes not technically successful because of significant hepatic vein thrombosis and inability to catheterize the hepatic vein. In these situations, direct intrahepatic portosystemic shunt (DIPS) is a viable alternative technique that can ameliorate portal hypertension in these patients.

In DIPS, a portocaval shunt is created between the inferior vena cava and the portal vasculature through the enlarged caudate lobe. The most crucial and difficult part of this procedure is identifying and gaining access to the portal vein. To address this issue, Haskal et al. first described the gun-sight technique in 1996 as method of guiding access into the portal vein [2]. Boyvat et. al then modified this technique and used transabdominal ultrasound guidance to percutaneously insert a needle into a portal venous branch and subsequently directly into the IVC [3]. Over the next two years, this simultaneous approach was technically and clinically successful in 11 patients [28]. Use of intravascular sonographic-guided placement of DIPS has been described by Petersen and Binkert [4, 29], wherein ultrasound is used to transhepatically puncture the portal vein from the IVC. A disadvantage of this approach is that it requires special equipment (endovascular ultrasound) and is therefore more expensive. Transabdominal ultrasound guidance with simultaneous fluoroscopy has been used successfully for intrahepatic puncture directly from the IVC to a portal venous branch [5]. In all cases described above, ultrasound guidance (both transabdominal and endovascular) was used to locate and target the portal vein for access.

In the case presented herein, ultrasound guidance was not used to generate the DIPS. Rather, a wedged hepatic venogram through catheterization of a thrombosed hepatic vein opacified the portal vein and provided a road map to guide access to the portal vein. In this approach, there are several important variables to consider. First, understanding the pathophysiology of Budd-Chiari syndrome is crucial to the success of interventional treatments, especially in DIPS. Because primary BCS is most often chronic in nature, several collateral pathways develop, varying based on the level of thrombosis and the span of the obstructed segment. Hepatic vein obstructions cause increased pressure within the hepatic venous system, resulting in formation of collateral venous circulation. However, the sites of these collateral veins in BCS are different from the portosystemic collaterals secondary to portal hypertension [30]. Multiple subcapsular vessels develop to bypass thrombosed portions of the hepatic veins as well as connect open portions of hepatic veins directly to systemic veins [30, 31]. This has been described as the pathognomonic “spiderweb” pattern on hepatic venocavography [9]. This existence of collaterals plays an important role in the course of BCS and must be considered during interventional treatment of BCS, as they may enable identification of the portal circulation and serve as a guide for DIPS.

In patients with Budd-Chiari syndrome, direct intrahepatic portosystemic shunt (DIPS) is a viable alternative technique to TIPS that can ameliorate portal hypertension. While DIPS generally involves the use of transabdominal or endovascular ultrasound to target the portal vein, the collateral vessels in BCS can be used to create a roadmap to facilitate targeting the portal vein. Importantly, long-term anticoagulation is needed in these patients to prevent Budd-Chiari recurrence and DIPS occlusion.
